# A new role of anterograde motor Kif5b in facilitating large clathrin-coated vesicle mediated endocytosis via regulating clathrin uncoating

**DOI:** 10.1038/s41421-018-0067-5

**Published:** 2018-12-25

**Authors:** Yan-Xiang Ni, Nan Zhou, Wen-Qian Xue, Li Rong, Wing-Ho Yung, Rao-Zhou Lin, Richard Yi-Tsun Kao, Zhi-Gang Duan, Hai-Tao Sun, Hua-Rui Gong, Xu-Ming Tang, Meng-Fei Liu, Wen Zhang, Shuang Qi, Sookja Chung, You-Qiang Song, Jian-Dong Huang

**Affiliations:** 1 School of Biomedical Sciences, The University of Hong Kong, Pokfulam, Hong Kong, SAR China; 2 State Key Laboratory of Brain and Cognitive Sciences, The University of Hong Kong, Pokfulam, Hong Kong, SAR China; 3 School of Biomedical Sciences, Faculty of Medicine, The Chinese University of Hong Kong, Shatin, NT, Hong Kong, SAR China; 4 Department of Microbiology, The University of Hong Kong, Pokfulam, Hong Kong, SAR China; 5 Department of Neurosurgery, Zhujiang Hospital, Southern Medical University, Guangzhou, China; 6 State Key Laboratory of Biopharmaceutical Biotechnology, The University of Hong Kong, Pokfulam, Hong Kong, SAR China; 7 Shenzhen Institute of Research and Innovation, The University of Hong Kong, Pokfulam, Hong Kong, SAR China; 8 Institute of Synthetic Biology, Shenzhen Institutes of Advanced Technology, Shenzhen, PR China

## Abstract

Kif5b-driven anterograde transport and clathrin-mediated endocytosis (CME) are responsible for opposite intracellular trafficking, contributing to plasma membrane homeostasis. However, whether and how the two trafficking processes coordinate remain unclear. Here, we show that Kif5b directly interacts with clathrin heavy chain (CHC) at a region close to that for uncoating catalyst (Hsc70) and preferentially localizes on relatively large clathrin-coated vesicles (CCVs). Uncoating in vitro is decreased for CCVs from the cortex of *kif5b* conditional knockout (mutant) mouse and facilitated by adding Kif5b fragments containing CHC-binding site, while cell peripheral distribution of CHC or Hsc70 keeps unaffected by Kif5b depletion. Furthermore, cellular entry of vesicular stomatitis virus that internalizes into large CCV is inhibited by Kif5b depletion or introducing a dominant-negative Kif5b fragment. These findings showed a new role of Kif5b in regulating large CCV-mediated CME via affecting CCV uncoating, indicating Kif5b as a molecular knot connecting anterograde transport to CME.

## Introduction

Anterograde intracellular transport and endocytosis, two opposite trafficking processes, contribute to plasma membrane homeostasis that is fundamental to membrane integrity, cell survival, and function. Whether and how these two trafficking processes communicate remain unknown, although feedback mechanism was found to perceive and respond to changes in lipid abundance on the plasma membrane^1^.

Clathrin-mediated endocytosis (CME) is a conserved and efficient way of reducing protein levels on the plasma membrane and maintains normal cellular functions^2–4^. It can also be hijacked by viruses, such as vesicular stomatitis virus (VSV), for entry into the host cells^5,6^. CME consists of highly coordinated steps, starting from the formation to the uncoating of clathrin-coated vesicles (CCVs). CCVs first assemble at the plasma membrane through the recruitment of a variety of cytosolic proteins in a highly regulated sequence^7^. Particularly, clathrin triskelia, composed by clathrin heavy chain (CHC), and light chains (CLC) are assembled into coat lattice surrounding the vesicle^8–11^. Uncoating then releases those proteins from CCVs back to the cytosolic pool, ensuring subsequent endocytic cellular events^12^. Both CCV assembly and uncoating are critical for progression of CME and the defect of either step can lead to impeded endocytosis^13–18^. Heat-shock cognate-70 protein (Hsc70), as an ATPase, serves as the major uncoating catalyst binding to the C-terminal tail of CHC (residues 1631–1675) on CCVs^19^. Through undergoing ATP hydrolysis, Hsc70 releases clathrin triskelia and coat proteins from CCVs^20,21^. The J cochaperone Auxilin, a cofactor of Hsc70, stimulates its ATPase activity, which facilitates clathrin uncoating^22–27^. Uncoating has also been reported to be regulated by modulation of auxilin or Hsc70 binding on clathrin triskelia^28,29^ or by other factors, such as synaptojanin^30^ and endophilin^18,31^. However, more intrinsic regulators contributing to CCV uncoating under cellular physiological conditions remain to be clarified.

Anterograde transport driven by kinesin-1, which consists of two heavy chains and two light chains (KLCs), delivers various proteins to cell periphery along microtubules and increases their levels on the plasma membrane^32^. The conserved and ubiquitous kinesin-1 heavy chain Kif5b contains a microtubule-interacting motor, a stalk region, a KLC-binding site, and a cargo-binding tail^32^. Kif5b is essential for the transportation of membranous organelles and vesicles^33,34^, including early endosomes^35,36^. After arriving at the cell periphery, Kif5b is released from microtubule and forms a folded and inactive conformation^37,38^. However, it is unclear if the released Kif5b plays any unknown role around the plasma membrane, e.g., regulating endocytosis.

Here, we provide evidence that anterograde motor Kif5b binds to the proximal segment of CHC, localizes on relatively large CCVs and plays a noncanonical role in CCV uncoating without affecting the distribution of CHC or Hsc70 at the cell periphery. We evaluated the effects of Kif5b depletion on CME and found that Kif5b depletion interfered with large CCV-mediated VSV cellular entry but hardly affected formation or function of synaptic vesicles. Furthermore, VSV entry was attenuated by applying a dominant-negative Kif5b fragment, which could overwhelm endogenous Kif5b for CHC binding in vivo. Overall, our study showed a new role of anterograde motor Kif5b in facilitating cargo specific-CME that involves large CCVs by regulation of clathrin uncoating.

## Results

### Kif5b is associated with CHC and localizes on relatively large mouse cortical CCVs

**Fig. 1:**
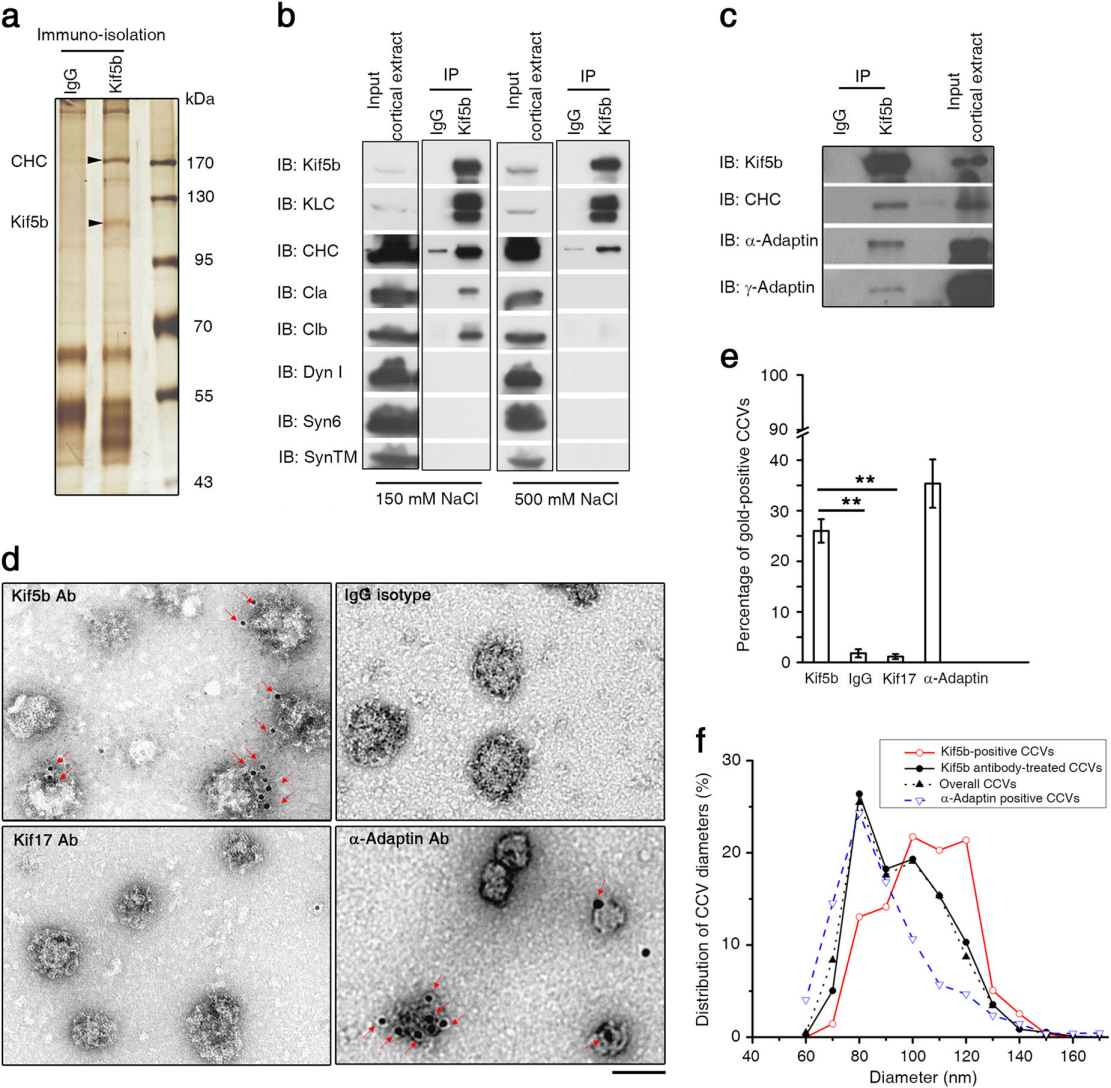
Kif5b is associated with CHC and localizes on relatively large mouse cortical CCVs. **a** Silver-staining analysis of immune-isolated mouse cortical extracts by using Kif5b antibody or control IgG. The lower and upper band (arrowheads) were verified as Kif5b and CHC by mass-spectrometry. **b**, **c** Immunoprecipitation of Kif5b in mouse cortical extracts. The precipitates were analyzed by indicated antibodies. **d** Representative electron micrographs of cortical CCVs stained with control IgG isotype or antibody against Kif5b, α-Adaptin or Kif17, followed by incubation of 2nd antibody conjugated to gold particles. Arrowheads indicate the gold particles representing Kif5b or α-Adaptin on CCVs. Scale bar = 100 nm. **e** Percentage of gold-positive CCVs in different samples. 305, 500, 324, and 1105 CCVs from images of 35, 46, 36, and 67 randomly-selected fields were analyzed for samples treated with Kif5b antibody, Kif17 antibody, control IgG isotype or α-Adaptin antibody, respectively. Error bars indicate s.e.m. **P < 0.01. **f** Diameter distribution of analyzed CCVs for different groups. Data of 933 CCVs from Kif5b antibody-treated samples, 277 Kif5b gold-positive CCVs, 385 α-Adaptin gold-positive CCVs and 2733 overall CCVs were used for plotting. IB immunoblot, IP immunoprecipitation, Cla Clathrin light chain A, Clb Clathrin light chain B, Dyn I Dynamin I, Syn6 Syntaxin 6, SynTM Synaptotagmin

To test whether Kif5b-mediated anterograde transport is linked to CME, we immuno-isolated Kif5b from mouse cortex and examined if any proteins involved in CME pathway were co-isolated. A ~170 kDa band was repeatedly detected and subsequently identified by mass spectrometry as CHC (Fig. 1a). To confirm the association of CHC with Kif5b, we immunoprecipitated Kif5b from mouse cortex under physiological (150 mM) as well as a more stringent (500 mM) salt condition and probed the co-precipitates by different antibodies. In addition to detecting KLC, we observed CHC in precipitates (Fig. 1b). Kif5b failed to co-precipitate syntaxin-6, synaptotagmin, or dynamin I, which were present in the cortical input, indicating the specificity of the co-immunoprecipitation assay. As CHC composes clathrin triskelion and serves as a core structural protein of CCV coat with AP-1 or AP-2 adaptor complex^12,39^, we further used co-immunoprecipitation to test whether Kif5b is associated with these adaptors besides CHC. α-Adaptin is a specific subunit of AP-2 localizing on endocytic CCVs^40^, whereas γ-Adaptin is a unique subunit of AP-1 on intracellular CCVs that travel between *trans*-Golgi and endosome^41^. We found that either α-Adaptin or γ-Adaptin was co-precipitated with Kif5b in mouse cortex extracts (Fig. 1c), suggesting the association of Kif5b with endocytic CCVs as well as non-endocytic ones travelling between *trans*-Golgi and endosome. The association of Kif5b with endocytic CCVs is particularly interesting and attracts our attention, since these vesicles are generally distributing at cell periphery where Kif5b is supposed to finish the transportation task and separated from its cargos.

To directly address if Kif5b localizes on cortical CCVs, we purified CCVs from mouse cortex and confirmed them by electron microscopy (EM) as electron-dense particles with characteristic CCV structures and diameter distribution^42–44^ (Supplementary Fig. S1). Then we performed immunogold EM with the purified CCVs using different primary antibodies and a gold-conjugated secondary antibody. As a positive control, α-Adaptin antibody efficiently recognized CCVs analyzed, as shown by the gold particles marked by red arrows (Fig. 1d, lower-right). Gold particles were also observed on CCVs treated with Kif5b antibody, rather than those with IgG isotype or antibody against other kinesin molecules, such as Kif17 (Fig. 1d, e), indicating Kif5b was specifically localizing on CCVs. The localization of Kif5b on cortical CCVs is likely relying on the specific interaction between Kif5b and CHC or CHC-binding proteins, as gel-enhanced liquid chromatography/tandem MS analysis (GeLC-MS/MS) of the cortical precipitates by Kif5b antibody detected CHC and CHC-associated proteins rather than other CME cargo proteins (e.g transferrin receptor) (Supplementary Table S1). When measuring the diameter profile of CCVs examined, we found that the group of CCVs recognized by α-Adaptin antibody shared with the overall purified CCV pool a similar diameter profile peaked at around 80 nm (Fig. 1f). Interestingly, the average diameter of CCVs recognized by Kif5b antibody shifted to ~100 nm (Fig. 1f). This indicates that Kif5b mainly localized on relatively large CCVs that contribute to 26 ± 2% of overall purified CCV pool (Fig. 1e). Thus, the above results demonstrate the specific localization of anterograde motor Kif5b on cortical CCVs, particularly the large ones.

### Kif5b directly interacts with CHC proximal segment via a 25-amino acid tail region

**Fig. 2:**
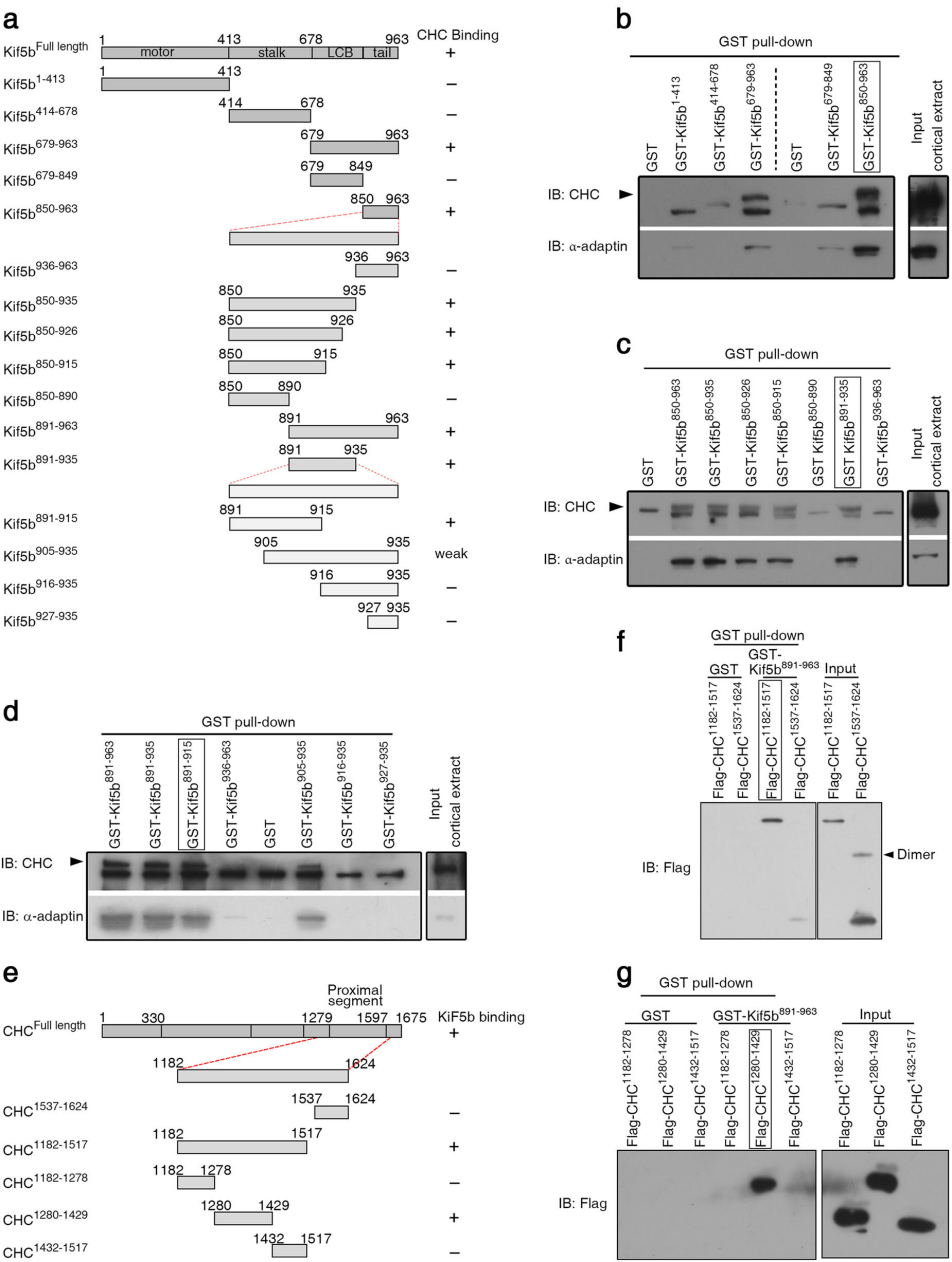
Kif5b directly interacts with CHC proximal segment via a 25-a. a tail region. **a** Schematic diagram of Kif5b fragments and their ability to bind CHC. LCB stands for light chain binding region. **b**–**d** Pull down of CHC and α-Adaptin in mouse cortices by different GST-fused Kif5b fragments (**b**) or tail truncations (**c**, **d**). Arrowheads indicates CHC above a non-specific band. **e** Schematic diagram of CHC fragments and their ability to bind Kif5b tail. **f**, **g** Pull down of recombinant flag-tagged CHC fragments from bacterial lysates by Kif5b^891-963^

To map the potential binding site on Kif5b for clathrin, we generated a series of Kif5b fragments that were fused to glutathione S-transferase (GST) (Fig. 2a). The fusion proteins were then immobilized and incubated with mouse cortical extracts. Next, we assessed the binding ability of different fragments by analysing the levels of co-precipitated CHC and α-Adaptin, since α-Adaptin is the marker of endocytic CCVs that we are interested. As shown in Fig. 2b, CHC and α-Adaptin were specifically pulled down by the Kif5b C-terminal fragment (residues 679-963) rather than the motor (residues 1-413) or stalk region (residues 414-678). The C-terminal fragment was further divided into two parts, Kif5b^679–849^ and Kif5b^850–963^. CHC and α-Adaptin were pulled down by Kif5b^850–963^, but not Kif5b^679–849^ containing a KLC-binding site (Fig. 2b), indicating a Kif5b-CHC association independent of KLC or KLC-Hsc70 interaction^45^. Further experiments with shorter truncations revealed that the tail (Kif5b^891–963^), Kif5b^850–915^ and Kif5b^891–935^ fragments were able to pull-down CHC or α-Adaptin, whereas Kif5b^850–890^ fragment could not (Fig. 2c), indicating that the CHC-binding site was localized within the 891–915 region of Kif5b. Indeed, it was found that the fragment Kif5b^891–915^ was sufficient to pull-down CHC and α-Adaptin (Fig. 2d). Collectively, these data revealed that the 25-amino acid tail region (residues 891–915) of Kif5b was a CHC-binding site and that Kif5b-CHC interaction was independent of KLC. We also studied the interaction between Kif5b^891–963^ and different recombinant fragments of CHC (Fig. 2e) and observed a direct interaction between CHC^1280–1429^, the N-terminus of CHC proximal segment^23,46^, and Kif5b tail (Fig. 2f, g). Taken together, these data demonstrated a direct interaction between residues 891–915 of Kif5b and N-terminus of the proximal segment of CHC.

### Kif5b plays a role in uncoating of mouse cortical CCVs without affecting the cell peripheral distribution of CCV coat proteins and uncoating catalyst

**Fig. 3:**
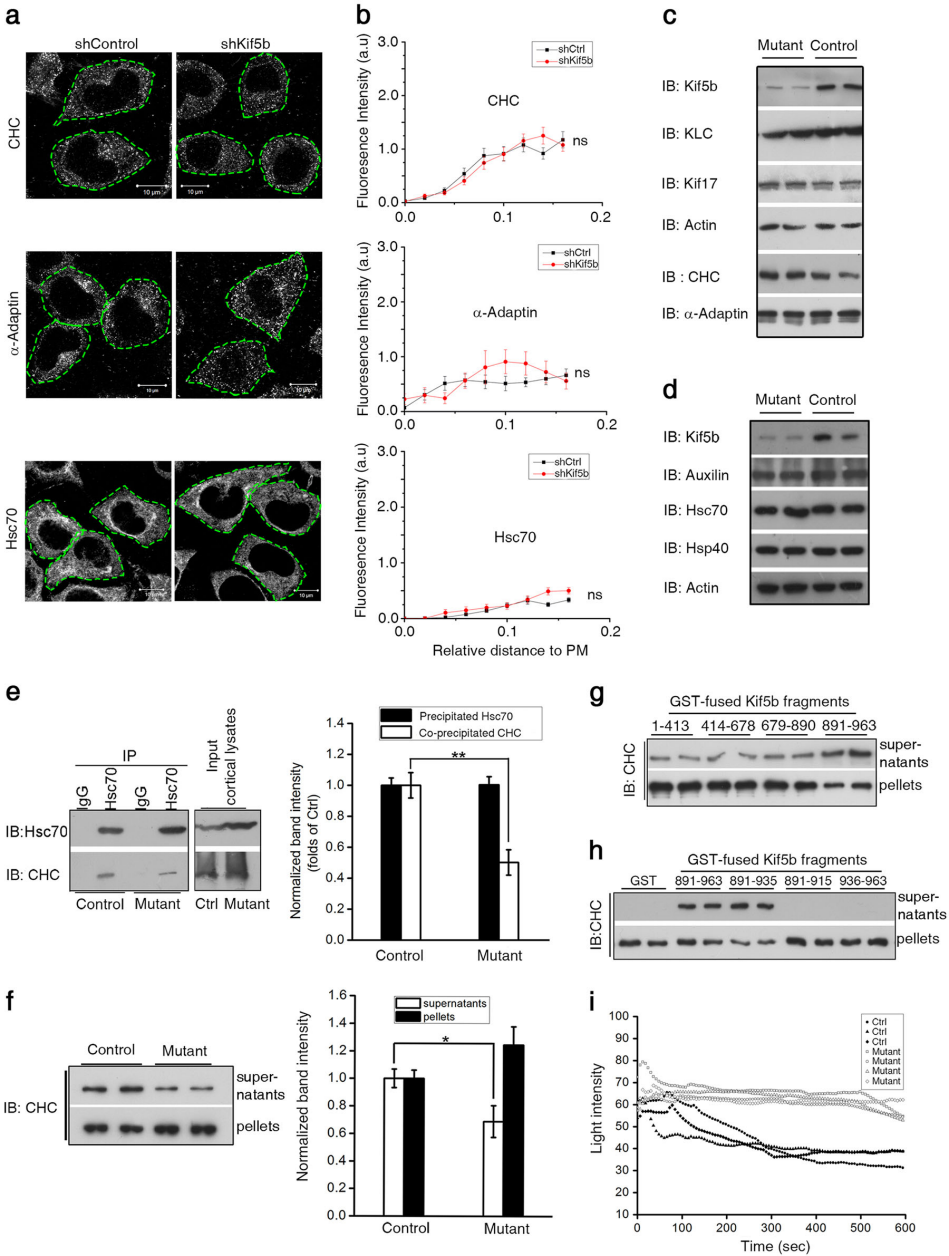
Kif5b plays a role in CCV coating without affecting cell peripheral distribution of CCV coat proteins and uncoating catalyst. **a** Representative immunofluorescence staining images for CHC, α-Adaptin, and Hsc70 in shKif5b or shControl RNA treated Neuro2a cells. Green dashed lines delineate the cell surface defined by co-staining of F-actin with phalloidion. Scale bar = 10 μm. **b** Quantitative analysis of subcellular distribution of CHC, α-Adaptin, and Hsc70. The distribution was quantified by measuring the line profile of fluorescence intensity from the plasma membrane (PM) to the nucleus (See also Supplementary Fig. S6). Error bars indicate s.e.m. *n* = 30. ns, not significant (*P* > 0.05). **c**, **d** Representative Western blots of cortical proteins or uncoating-related proteins in cortices of *kif5b* mutant mice or control littermates. **e** Left column: immunoprecipitation of Hsc70 in *kif5b* mutant mouse cortices or controls. The precipitated Hsc70, co-precipitated CHC or input cortical lysates were subjected to Western blot analysis. Right column: quantitative analysis of bands of precipitated Hsc70 and co-precipitated CHC from mutant cortices (*n* = 4) and controls (*n* = 4). Error bars indicate s.e.m. **P < 0.01. **f** left column: uncoating assay of CCVs from mutant or control mouse cortices. The amount of CHC released into supernatants was representative of uncoating. Right column: quantitative analysis of released CHC. Error bars indicate s.e.m. *n* = 3. *P < 0.05. **g**, **h** Uncoating assay of CCVs treated with or without different recombinant fragments of Kif5b. **i** Representative disassembly curve of purified cortical CCVs from mutant or control mice detected by light scattering assay in vitro

Given that Kif5b interacts with CHC and localizes on endocytic CCVs marked with α-Adaptin, we wondered if this well-known anterograde motor is responsible for the intracellular trafficking of components of endocytic CCVs. To address this question, we knocked down Kif5b in Neuro2a cells with the use of shRNA (Supplementary Fig. S2a, b). Immunofluorescence staining and quantitative analysis revealed that the abundance of CHC, α-Adaptin, and Hsc70 in cellular compartments close to plasma membrane remained almost unchanged in Kif5b-depleted cells (Fig. 3a, b). These results suggest that cell peripheral distribution of major CCV components and uncoating catalyst is likely independent of Kif5b.

To further investigate potential new function(s) of Kif5b on CCVs, we employed a *kif5b* conditional knockout (mutant) mouse (*Camk2a-cre; kif5b*^*-/floxP*^, Supplementary Fig. S2c) that expressed Cre in the forebrain^47^. In cortices of mutant mice, Kif5b expression was decreased by more than 60% compared with controls, whereas other kinesin proteins (e.g., KLC and Kif17) remained almost unchanged (Fig. 3c, Supplementary Fig. S3a). We also examined the expression of CCVs proteins and found that levels of coat proteins (e.g., CHC and α-Adaptin) and CCV uncoating-related proteins (Hsc70, cofactors Hsp40, and Auxilin) were not affected in cortices of mutant mice (Fig. 3d, Supplementary Fig. S3b). The above findings implied that Kif5b is possibly involved in other cellular function(s) instead of the anterograde transportation of CCV proteins. Notably, when testing the in vivo CHC-Hsc70 interaction that is important for CCV uncoating by immunoprecipitating mice cortical lysate with Hsc70 antibody, we detected less CHC in Hsc70 precipitates from mutant mice than from controls, although similar amounts of Hsc70 were precipitated from both samples (Fig. 3e). This decreased binding between Hsc70 and CHC in mutant mice implied the involvement of endogenous Kif5b in modulating CHC-Hsc70 interaction and possibly CCV uncoating.

We then tested whether Kif5b could directly affect CCV uncoating under physiological conditions. To this aim, we modified an established centrifugation-based uncoating assay^20,21^. After ultracentrifugation, disassembled CCVs released coat proteins into the supernatant, whereas intact CCVs and uncoated vesicles remained in the pellet. Significantly less CHC was released into the supernatant from mutant CCV reactions than that from control reactions, even though the input CCVs were of the same amount (Fig. 3f), indicating Kif5b was involved in CCV uncoating. Next, we added different GST-fused Kif5b fragments (Supplementary Fig. S4a) into uncoating reactions. Addition of Kif5b tail that carries the CHC-binding site could increase CHC release, but not for other fragments without CHC-binding site, including the motor (Kif5b^1–413^), stalk region (Kif5b^414–678^), and KLC-binding site-containing fragment (Kif5b^679–890^) (Fig. 3g). In addition, the Kif5b tail fragment Kif5b^891-963^ could cause a dose-dependent increase of CHC release in the uncoating reaction (Supplementary Fig. S4b, c). These results indicated the requirement of Kif5b-CHC interaction for uncoating. We further analyzed the effects of various tail regions and observed that uncoating was facilitated by Kif5b^891–935^ similar to the tail Kif5b^891–963^ whereas the minimal CHC-binding region Kif5b^891–915^ was not able to facilitate uncoating (Fig. 3h, Supplementary Fig. S4d). Notably, the requirement of Kif5b-CHC interaction for uncoating was further proved by using a real-time uncoating assay of light scattering intensity that dynamically reflects vesicle diameter change^48,49^. We found that CCVs from mutant mouse cortices exhibited a slower decrease of light scattering intensity than those from control samples during the 10-min reaction period (Fig. 3i). Overall, these data showed that anterograde motor Kif5b plays a role in regulating the uncoating of mouse cortical CCVs.

### Large CCV-mediated endocytosis of VSV is inhibited by Kif5b depletion or a dominant-negative Kif5b fragment for uncoating

The formation and uncoating of CCVs are highly coordinated to guarantee smooth and continuous CME. Over 50 cytosolic proteins participate in CCV formation^7^, and efficient uncoating is required to recycle those proteins for new budding endocytic vesicles to ensure next rounds of endocytosis. When uncoating is impeded, some endocytic factors might become limiting for CCV formation, although major CCV coat proteins (e.g., CHC) are abundant in the cytosolic pool. Thus, besides direct defects in endocytic CCV formation, interference of late step of endocytosis like uncoating can also lead to disordered CME^16–18,24,29^. For example, abolishing of GRK2-dependent Ser204 phosphorylation on clathrin light chain b (CLCb) caused the reduced endocytosis of a subset of G protein-coupled receptors (GPCRs) by slowing down clathrin uncoating^29^. Besides, loss of inositol 5-phosphatase (OCRL) in Lowe syndrome patient fibroblasts caused inefficient uncoating and subsequently resulted in an endocytosis defect^16^. Since Kif5b was found to be involved in CCV uncoating, we then asked if Kif5b will also consequently regulate CME.

The sizes of CCVs are known to vary between species or, within the same species, depend on cargoes or different cell types^2,11,50^. As Kif5b was found to preferentially bind to relatively large CCVs, we expect that Kif5b will not affect endocytic pathway globally, instead, it may function in CME in a CCV-size dependent manner. To test this possibility, we first examined whether Kif5b depletion affected the formation of synaptic vesicles, which are of small size (40–45 nm in diameter) resulted from coated vesicle disassembly^8,51,52^. EM examination of synaptic vesicles in mouse cortex revealed no obvious accumulation of coated structures in the synapses from *kif5b* mutants compared to control mice (Supplementary Fig. S5a). We also performed whole-cell patch-clamp recordings of pyramidal neurons in 20-day-old mouse hippocampal sections, in which the Kif5b expression was remarkably down-regulated by *kif5b* knockout (Supplementary Fig. S5b, c). Between mutant and control sections, we observed no significant differences in amplitude or frequency of miniature inhibitory postsynaptic currents (mIPSCs, Supplementary Fig. S5d) nor miniature excitatory postsynaptic currents (mEPSCs, Supplementary Fig. S5e). These lines of morphological and functional evidence revealed no changes of synaptic vesicle formation or related functions in *kif5b* mutants.

**Fig. 4:**
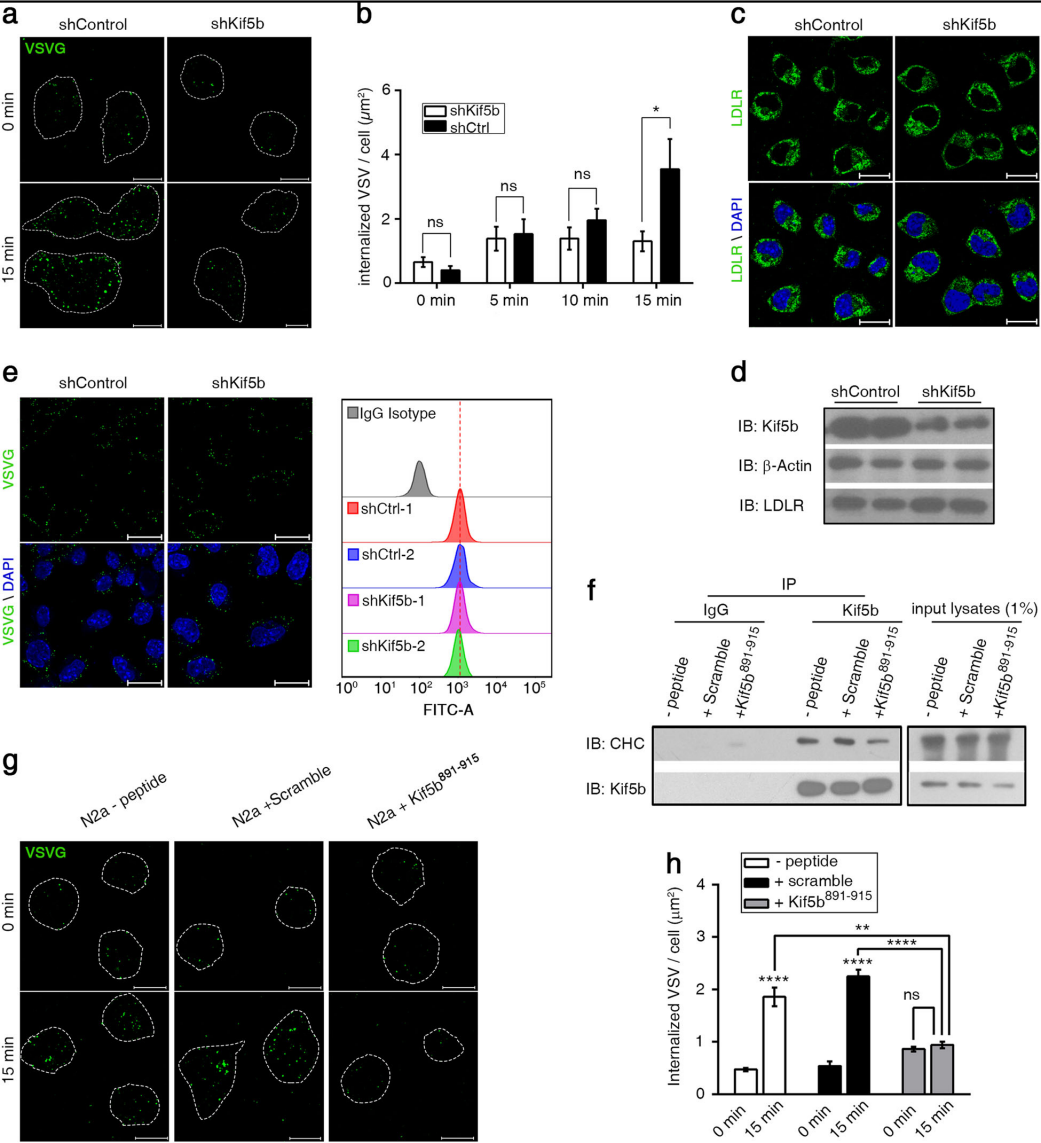
Kif5b regulates large CCV-dependent VSV cellular entry. **a** Representative immunofluorescence staining images of internalized VSVs in shKif5b or shControl Neuro2a cells after 0 or 15 min VSV infection. White dashed lines delineate the cell surface defined by co-staining of F-actin with phalloidion. Green pseudo color was used for VSV signals. Scale bar = 10 μm. **b** Quantitative analysis of internalized VSV signals for shKif5b or shControl Neuro2a cells infected by VSV with different time slots. **c** Representative immunofluorescence staining images for LDLR (green) in shKif5b or shControl RNA treated Neuro2a cells counterstained with DAPI (blue). Scale bar = 20 μm. **d** Western blot analysis to compare LDLR expression level in shKif5b or shControl RNA treated Neuro2a cells. **e** left column: representative immunofluorescence staining images of VSVs (Green) bound to cells counterstained with DAPI (blue). Scale bar = 20 μm. Right column: quantification of binding of VSVs to shKif5b or shControl RNA treated Neuro2a cells by FACS flow cytometry. Red dashed line indicates similar fluorescence value among histograms of different experimental groups. Neuro2a cells bound with viruses were stained with normal IgG isotype as a negative control. **f** Co-immunoprecipitation of CHC with Kif5b from Neuro2a cells with 1 h pre-treatment with or without 100 µM cell-penetrating scramble or Kif5b^891-915^ peptide at 37 °C. **g** Representative immunofluorescence staining images of internalized VSVs in Neuro2a cells with 15 min infection after 1 h pretreatment without peptide or with 100 µM scramble or Kif5b^891–915^ peptide at 37 °C. Scale bar = 10 μm. **h** Quantitative analysis of internalized VSV signal in different Neuro2a cell groups indicated in (**g**). Error bars indicate s.e.m. *n* > 30. ****P < 0.0001; ** *P* < 0.01; *P < 0.05. ns, not significant (*P* > 0.05)

To further test whether Kif5b regulates CME that involves large size CCVs, we adopted VSV cellular internalization as our model here. Although many cell-surface receptors and viruses enter cells via CME, VSV internalizes through large CCVs with a dimension of about 180 × 70 nm during infection^53^. We applied replication-deficient VSV pseudoparticles and infected cells for different time slots, followed by immunofluorescence staining of all internalized viruses in cells with the use of a VSV-specific antibody (Fig. 4a). Quantitative analysis showed a remarkable accumulation of internalized VSVs along infection time in shControl cells but not in shKif5b cells (Fig. 4b). A significant difference was observed between the two groups after 15 min infection (Fig. 4b). These data suggested that Kif5b regulates large CCV-dependent cellular entry of VSV.

LDL receptor (LDLR) serves as the major entry port of VSV in mammalian cells^54^. An alternative explanation for the observed reduction of VSV internalization in Kif5b-depleted cells could be due to altered distribution or expression of LDLR and consequently decreased virus binding to cellular plasma membrane. To test this hypothesis, we compared the cellular distribution or expression of LDLR in shControl and shKif5b cells. Immuofluoresence staining and Western blot showed that both of them were not altered by Kif5b depletion (Fig. 4c, d). We also tested the cellular binding affinity of VSV by incubating VSV with shControl and shKif5b cells. The surface-bound virus was examined by immunofluoresence staining and also quantitatively analyzed through flow cytometry. The data showed that the Kif5b-depleted cells exhibited no defects in binding to VSV compared to control cells (Fig. 4e). Together, these results showed that the reduced VSV uptake in Kif5b-depleted cells is not due to altered virus binding before endocytic events started.

To directly confirm the aforementioned regulation of VSV internalization is contributed by the new role of Kif5b in CCV uncoating, we applied a TAT-conjugated^55^ cell-penetrating peptide corresponding to Kif5b^891–915^, which was shown to bind to CHC without facilitating CCV uncoating in vitro (Fig. 2d and Fig. 3h) and is expected to act as a dominant-negative fragment for uncoating in vivo. A dominant-negative approach typically leads to loss of function of a wild-type protein through the sequestration of its effectors. Consistently, we found that less CHC was co-immunoprecipitated with Kif5b in cells pretreated with Kif5b^891–915^ peptide than in controls without any peptide pre-treatment or with a scramble peptide possessing same amino acids composition as Kif5b^891–915^ (Fig. 4f), suggesting that Kif5b^891–915^ could overwhelm endogenous Kif5b for CHC binding in vivo. We then assessed the effect of peptide pretreatment on VSV cellular entry. Immunofluorescence staining and quantitative analysis of internalized VSVs in cells with 15 min-VSV infection showed a significant decrease of VSV entry in the cell group pretreated with Kif5b^891–915^ compared with the control groups (Fig. 4g, h). Overall, these data showed that Kif5b regulates VSV cellular entry depending on its role in uncoating.

## Discussion

Various mechanisms contribute to the maintenance of plasma membrane homeostasis, including coordination between different endocytic pathways^56^. In this study, we observed the localization of Kif5b on large CCVs at the N-terminal part of CHC proximal segment, which is spatially near the Hsc70-binding tripod on CCVs^23,46,57^. Depletion of Kif5b impeded CCV uncoating that is essential for efficient CME. Therefore, Kif5b may serve as a molecular knot, contributing to the connection between anterograde transport and CME, and the potential feedback loop for plasma membrane homeostasis. These findings provide a possible explanation for how cells can partially protect plasma membrane integrity against anterograde transport fluctuations: as kinesin-1-mediated anterograde transport delivers more proteins to the plasma membrane, more Kif5b will reach the cell periphery to increase CME; conversely, when kinesin-1-mediated anterograde transport slows down, less Kif5b is available to CCVs, thus decreasing CME.

CCV uncoating has been intensively studied by using recombinant clathrin cages generated in vitro, especially when dissecting the mechanism of how a known uncoating regulator, such as Hsc70, conveys its effect^23,46,48^. Here, to address Kif5b as a new intrinsic uncoating regulator, we applied purified cortical CCVs instead of recombinant clathrin cages and chose not to add any extra factors like Hsc70 or Auxilin into the reaction. This approach maintained the ratio of different proteins on the assayed CCVs largely similar to that under physiological conditions, which avoided masking the undetermined function of Kif5b. In these reactions, uncoating are facilitated by Kif5b fragments (e.g., residues 891–963 or residues 891–935) containing the CHC-binding site rather than by fragment 936–963 without CHC-binding site, indicating that the Kif5b-CHC specific interaction is indispensable for uncoating function of Kif5b. Given the unaltered cell peripheral distribution and expression level of Hsc70 (Fig. 3a–d), but decreased Hsc70-CHC interaction in *kif5b* mutant samples (Fig. 3e), the specific Kif5b-CHC binding may affect CCV uncoating via modulating Hsc70-CHC interaction. Indeed, regulatory mechanism of uncoating through modulation of Hsc70 activation or binding to CCVs was proposed recently^28^. In addition to the potential mechanism of modulating Hsc70-CHC interaction, the physical binding of Kif5b on CCVs may also directly increase the strain on the coat and aggravate Hsc70-mediated uncoating. In the collision-pressure model proposed to illustrate Hsc70 force generation during clathrin-coat disassembly, Hsc70 made CCVs rigid but prone to catastrophic deformation (disassemble) when probed with an external strong force^58^. Thus, binding of excess Kif5b on CCVs may augment the external force and facilitate the disassembly of clathrin-cage. This may also explain why CHC-binding Kif5b fragments could facilitate CCV uncoating in a dose and mass dependent manner (Supplementary Fig. S4b, Fig. 3h): more CHC-binding fragments added can generate stronger force on the structural wall, while binding of the minimal Kif5b fragment (Kif5b^891–915^) with low molecular mass cannot produce enough force to facilitate disassembly.

Kif5b has been repeatedly observed to preferentially localize on relative large CCVs with an average vesicle diameter around 100 nm (Fig. 1f). Such preference is possibly because large and small CCVs are different from each other in various aspects. For example, the formation of large CCVs requires actin filaments while that of conventional small ones do not^53,59^. In addition, although large and small CCVs share many common coat proteins (e.g., CHC), there might be some intrinsic factors specifically residing on large CCVs^60–62^ and facilitating Kif5b-CCV association, since unique components have been observed in large CCVs in our CCV silver-staining experiment (data not shown). Moreover, large CCVs generally showed longer lifetime^6,53,61^, which may also offer a better chance for cytosolic Kif5b to detect and localize on them. Cellular CCVs have great diversity in terms of size and the cargoes they carried^2,11,50^. Differential regulatory mechanisms may exist for modulating the uncoating of specialized CCV populations. For example, CME of a subset of G protein-coupled receptors (GPCRs) whose endocytosis is GRK2-dependent required the phosphorylation of CLCb. The phosphorylation enhanced the rate of uncoating of those CCVs, possibly contributing to intracellular signaling of particular GPCRs^29^. Moreover, a stabilized and long-lived pool of AP-2 CCV in synapses was reported. The stabilization of those CCVs were mediated by differential molecular mechanisms of decreased binding Hsc70 and synaptojanin1 and enhanced μ2/AP-2 phosphorylation and activation^28^. Thus far, we proposed a specialized CCV pool of relatively large vesicle size, the efficient uncoating of which involves Kif5b as an additional intrinsic regulator. Large CCVs typically contain more coat components and have longer lifetime^6,53,61^. Additional requirement of Kif5b binding may facilitate their rapid uncoating to ensure subsequent rounds of endocytic events. In line with this observation, Kif5b depletion could specifically interfere large CCV-dependent VSV uptake (Fig. 4a, b) rather than the formation and function of synaptic vesicle (Supplementary Fig. S5), the internalization of which relies on CCVs of relatively small size. In our study, introducing a 25-amino acid dominant-negative Kif5b fragment in uncoating decreased VSV cellular entry (Fig. 4g, h), indicating the potential application of CHC-binding peptide in impeding VSV infection. Therefore, the Kif5b-CHC interaction might serve as a target for developing anti-viral drugs without side effects on normal cellular functions such as synaptic transmission.

## Materials and methods

### Reagents, antibodies, and plasmids

Protease inhibitor cocktail (Sigma, 4693132001) and protein G-Agrose (Roche, 11719416001), and antibodies against KLC (Chemicon, MAB1616), Kif17 (Sigma, K3638), CHC (BD Biosciences, 610500), Cla (Santa Cruz), Clb (Santa Cruz), Hsc70 (Santa Cruz, sc7298 and Enzo, ADI-SPA-816-D), α-Adaptin (BD Biosciences 610502), γ-Adaptin (BD Biosciences 610386), Dynamin I (Santa Cruz, sc-12724), Syntaxin 6 (BD Transduction Laboratories, 610636), Synaptotagmin (BD Transduction Laboratories, 610433), EEA1 (UPSTATE, 07-292), Auxilin (Santa Cruz, sc104213), Hsp40 (Enzo Life Sciences, ADI-SPA-400-D), Beta Actin (Sigma, A5316), and VSV-G (Abcam, ab1874) were used. The antibody against Kif5b was previously described^63,64^. The following secondary antibodies for immunofluorescence staining were used: Cy3 donkey anti-mouse (Jackson Immunoresesarch), Cy3 donkey anti-rabbit (Jackson Immunoresesarch), Alexa Fluor 488 donkey anti-mouse (Jackson Immunoresearch) and Alexa Fluor 488 donkey anti-rabbit (Jackson Immunoresearch). Cell-penetrating peptides synthesized in GL biochem (Shanghai) Ltd are: TAT-Kif5b^891–915^ peptide GRKKRRQRRRPPQDRKRYQQEVDRIKEAVRSKNMARRG and the control TAT-scramble peptide: GRKKRRQRRRPPQRDVRDRGANKIQAREYRQRKVMESK. The GST-fused Kif5b fragments were generated by PCR amplification of pCDNA3.Kif5b plasmid and subcloned into pGEX-4T-1 (Novagen).

### Cell lines and animals

The cell lines used in this study are Neuro2a neuroblastoma cells and 293T human embryo kidney cells. The *kif5b*^*+/*^^*−*^ and *kif5b*^*floxP/floxP*^ mice were generated by gene targeting as described previously^63^. To obtain a conditional knockout mouse, *kif5b*^*+/−*^ mouse was first crossed with a Camk2a-cre transgenic line^47^ to generate a *kif5b*^*+/*^^*−*^; *Camk2a-cre* mouse, which was subsequently bred with *kif5b*^*floxP/floxP*^ to get the final conditional knockout mice (*kif5b*^*−*^^*/floxP*^*; Camk2a-cre*) as well as their littermates (*kif5b*^*+/floxP*^). All animal experimentation was approved and performed in accord with the guidelines of the Committee on the Use of Live Animals in Teaching and Research at the University of Hong Kong regarding the care and use of laboratory animals.

### Immunoprecipitation

Mouse cortices were lysed in lysis buffer (50 mM Tris pH 7.4, 150 mM NaCl, 2 mM EDTA, 1% Triton X-100, Protease inhibitor cocktail), followed by centrifugation at 15,000 × *g* for 15 min. Supernatants were then incubated with antibodies and protein G-Agarose. The bound vesicles/proteins were washed several times. Samples were eluted in SDS-PAGE sample buffer at 55 °C, and subjected to SDS-PAGE or Western blot analysis using the indicated antibodies. Enhanced chemiluminescence detection was performed using SuperSignal West Pico reagent (Pierce, 34577). Intensities of the bands were quantified by ImageJ (NIH).

### Liquid chromatography/tandem MS analysis

Biological triplicated samples were applied for electrophoresis using NuPAGE® Novex® 4–12% bis-tris protein gels. In-gel digestion was performed following an optimized protocol^65^. Nanoflow electrospray ionization tandem mass spectrometric analysis of peptide samples was carried out using LTQ-Orbitrap Velos (Thermo Scientific, Bremen, Germany) interfaced with Agilent’s 1200 Series nanoflow LC system.

### Pull-down assay

GST-fused Kif5b fragments and Flag-tagged fragments of CHC were expressed in BL21 cells at 18 ℃ and prepared at 4 ℃. The GST-fusion proteins were subsequently immobilized on glutathione-Sepharose beads (GE Healthcare Life Sciences, 17075605) and eluted with 0.2 M glutathione (pH 8.0) for the uncoating reaction or incubated with mouse cortical or bacterial extracts containing flag-tagged CHC fragment in lysis buffer (50 mM Tris pH 7.4, 150 mM NaCl, 2 mM EDTA, 1% Triton X-100, Protease inhibitor cocktail) for the pull-down assay.

### Immunofluorescence microscopy

Neuro-2a cells cultured on glass coverslips were washed by PBS twice, fixed in 4% paraformaldehyde (PFA) in PBS for 10 min, permeabilized in 0.25% Triton X-100 in PBS for 10 min, and incubated with blocking buffer (5% donkey serum in PBST) for 30 min at room temperature. Cells were incubated with indicated primary antibodies overnight at 4 ℃, followed by incubation with fluorophore-conjugated secondary antibodies for 1 h at room temperature. Coverslips were then mounted onto glass slides with SlowFade™ Gold Antifade Mountant with DAPI (Life Technologies, S36938). Images were acquired by Zeiss LSM780 confocal laser scanning microscope. Images were processed by ZEN Lite (ZEISS).

### Lentivirus generation and infection

A modified pLL3.7/U6 promoter vector with puromycin resistance was used to express Kif5b shRNA targeting the murine kif5b-encoded mRNA (GenBank accession number for murine Kif5b: NM_008448.3). Two shRNA sequences were selected to target murine Kif5b: 5'- GGACAGATGAAGTATAAATTTCAAGAGAATTTATACTTCATCTGTCC-3' and 5'-GGCTCTTTCTATTATATCATTCAAGAGATGATATAATAGAAAGAGCC-3'. The control sequence did not target mRNA of any genes: 5'- GACTACCGTTGTATAGGTG TTCAAGAGA CACCTATACAACGGTAGTC-3'. To generate the lentivirus, 2 × 10^6^ 293T cells were co-transfected with 10 μg of the specified pLL lentiviral vector and 3.3 μg of each of the packaging vectors (pMD2G-VSVG, pRSV-REV, and pMDL g/p RRE) by the calcium phosphate precipitation method. After 48 h transfection, the supernatant from the transfectant was collected and filtered through 0.45-μm filters (Corning). The medium of the Neuro2a cells was replaced with virus-containing supernatant supplemented with 8 μg/ml polybrene (Sigma, 107689) and incubated for 24 h. Puromycin at a final concentration of 1 μg/ml was added to the culture medium 48 h after transduction.

### CCV purification and EM analysis

CCVs were purified from mouse cortices according to modified protocols^42–44^. Mice cortices were homogenized in Mes buffer (100 mM MES, 1 mM EGTA, 2 mM MgCl_2_, pH 6.5) and centrifuged at 1000 × *g* for 10 min at 4 ℃. The resultant supernatant was layered onto a 5% glycerol pad and centrifuged at 100,000 × *g* for 1 h. The pellet was re-suspended, and mixed 1:1 with Mes buffer containing 12.5% Ficoll 400 and 12.5% sucrose and centrifuged at 40,000 × *g* for 40 min. Supernatant was diluted 1:5 with Mes buffer, filtered in 0.2-µm filters (Corning, 431224), and centrifuged at 33,000 × *g* for 1 h. The CCV-containing pellet was re-suspended for further use. For EM analysis, enriched CCVs were fixed with 4% PFA and absorbed into carbon-coated formvar grids. The grids with CCVs for gold staining were stained overnight at 4 ℃ with control IgG isotype, Kif5b antibody, α-Adaptin or Kif17 antibody, followed by 1 h incubation of 10-nm gold-conjugated anti-rabbit or 12-nm gold-conjugated anti-mouse second antibody (Jackson Immunoresearch). Negative staining was performed with 2.0% uranyl acetate for 10 s at room temperature. Electron micrographs were collected from randomly-selected fields using Philips EM 208S (Philips). The images were analyzed by a blind analysis.

### Centrifugation-based CCV uncoating

The uncoating assay was modified from established protocols^20,21^. The CCVs (120 µg) were incubated for 8 min at 25 °C in the absence or presence of the indicated fusion proteins (20 µg) in a total volume of 500 µl containing 2 mM ATP, 75 mM KCl, 5 mM MgCl_2_, and protease inhibitor cocktail. The reaction mixtures were centrifuged at 100,000 × *g* for 10 min at 4 °C. Supernatants and pellets were subjected to Western blot analysis.

### Light scattering-based CCV uncoating

Equal amount of purified CCVs from mutant or control mice was mixed at 4 ℃ with 1 mM ATP in a total volume of 30 μL in buffer (40 mM HEPEs, pH 7.0, 75 mM KCl, 4.5 mM Mg acetate). The mixtures were added to 384-well black polystyrene plate (Sigma, CLS3702-25EA) on the ice and spun down for 5 s to remove any bubbles inside the reaction. Light scattering^48,49^ was immediately monitored using Wyatt DynaPro at 25 ℃ every 3 s for up to 600 s.

### VSV pseudoparticle binding assay

Aliquots of viral dilutions produced from transfection of HEK293T cells with VSV pseudoparticle packaging vectors were incubated with Neuro2a cells for 1 h at 4 ℃. After extensive washing with cold PBS to remove unbound viruses, cells were immediately fixed with 4% PFA for 15 min at room temperature. Bound viruses were examined by immunofluorescence staining with antibody against VSV-G and Alexa Fluor 488 donkey anti-rabbit secondary antibody. The amount of bound viruses was quantitatively measured by flow cytometer (BD LSR Fortessa). Data was analyzed by FlowJo.

### VSV pseudoparticle internalization assay

Aliquots of viral dilutions produced from transfection of HEK293T cells with VSV pseudoparticle packaging vectors were incubated with Neuro2a cells for 1 h at room temperature. After washing by DMEM, cells were shifted to 37 ℃ for indicated time for virus infection. Internalized VSVs were fixed and detected by an antibody against VSV-G. Intracellular VSV signals were blindly analyzed by the Particle Analyze function of ImageJ with a uniformly defined image threshold. Background signal noise calculated from cell groups without VSV treatment was subtracted.

### Statistical analysis

All data are expressed as mean ± s.e.m. Student's *t*-test (unpaired) was used to compare two groups (*P* < 0.05 being considered significant).

## Electronic supplementary material

Supplementary Information

## Electronic supplementary material

**Supplementary Information** accompanies the paper at (10.1038/s41421-018-0067-5).
