# Supplementary material for: A new role of anterograde motor Kif5b in facilitating large clathrin-coated vesicle mediated endocytosis via regulating clathrin uncoating
Source: Cell Discov. 2018 Dec 25;4:65. doi: 10.1038/s41421-018-0067-5 (PMC6305376; doi:10.1038/s41421-018-0067-5)
Supplement: Supplementary file 1 — Supplementary Information [file 41421_2018_67_MOESM1_ESM.pdf]

Supplementary Information

A new role of anterograde motor Kif5b in facilitating large clathrin-coated vesicle mediated endocytosis via regulating clathrin uncoating

Yan-Xiang Ni[1,4*], Nan Zhou[1*], Wen-Qian Xue[1*], Li Rong[1], Wing-Ho Yung[2], Rao-Zhou Lin[1], Richard Yi-Tsun Kao[3], Zhi-Gang Duan[1,4], Hai-Tao Sun[1,8], Hua-Rui Gong[1], Xu-Ming Tang[1], Meng-Fei Liu[1], Wen Zhang[1], Shuang Qi[1], Sookja Chung[1,5], You-Qiang Song[1] and Jian-Dong Huang[1,4,6,7#]

Figure S1

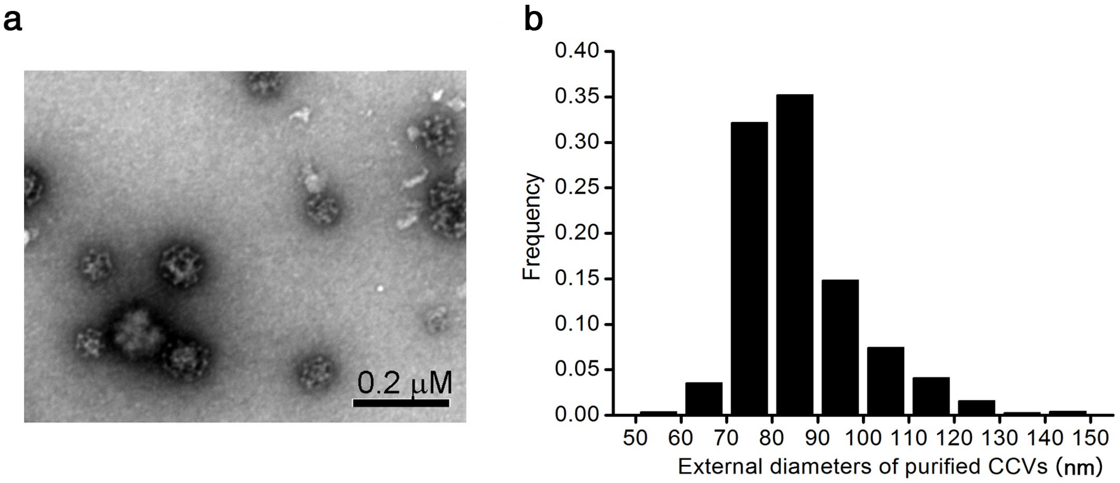

Figure S1. Confirmation of the purified cortical CCVs by negative staining and electron microscopy, related to Figure 1. (**a**) CCVs were purified from mice cortices and examined by electron microscopy after negative staining. The purified CCVs preserve their typical coat structures well with application of the ice-cold acidic Mes buffer. Scale bar = 0.2 μm. (**b**) External diameter distribution of purified cortical CCVs measured from high-magnification electron micrographs.

Figure S2

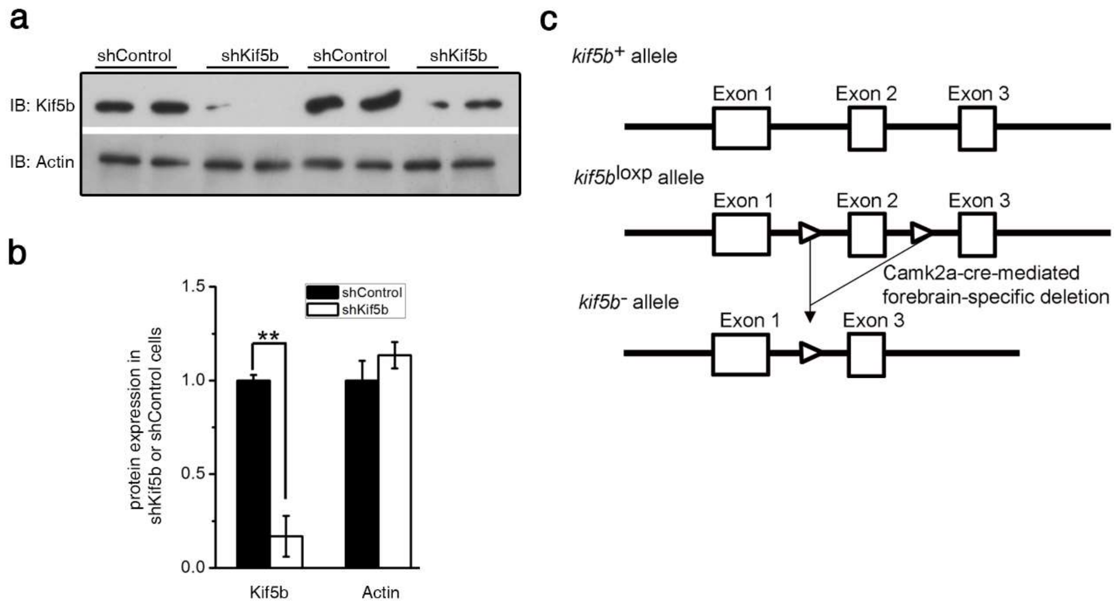

Figure S2. Kif5b knock-down by shRNA and *kif5b* conditional knock-out in mouse cortex, related to Figure 2. (**a**) Kif5b depletion in Neuro2a cells by shRNA was confirmed by Western blot analysis with indicated antibodies. (**b**) Quantitative analysis of the indicated bands in (a). The protein expression levels in shControl cells were normalized to 1.0, and the levels of the proteins in shKif5b samples were expressed as the relative to the normalized values. Error bars indicate s.e.m. $**P<0.01$ as analyzed by Student's t-test. (**c**) Schematic illustration of Camk2a-cre-mediated deletion of exon 2 from the *kif5b* allele.

Figure S3

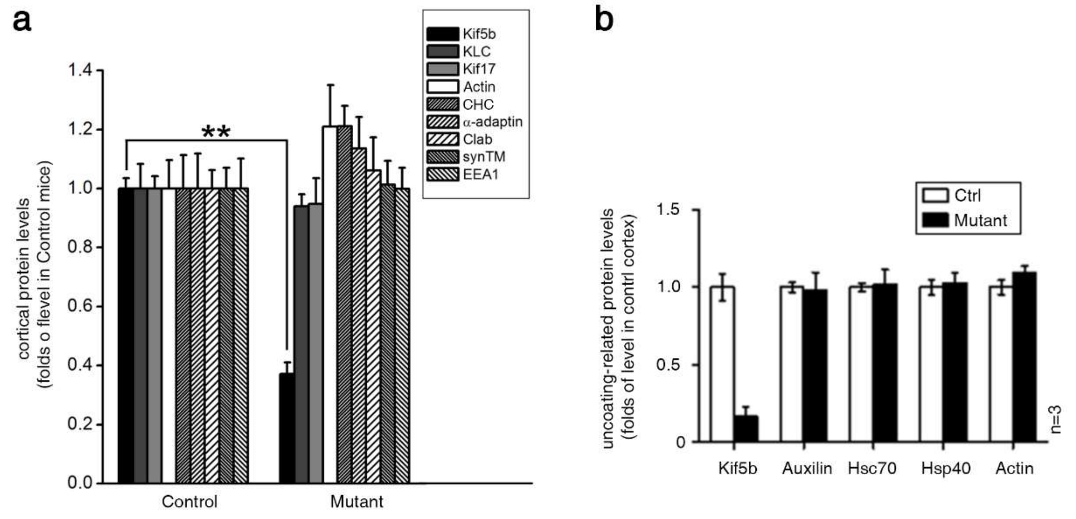

Figure S3. Quantitative analysis of cortical protein and uncoating related protein levels in the mutant or control mouse cortex, related to Figure 3. (**a**) Quantitative analysis of the indicated cortical protein levels (Fig. 3c) from *kif5b* mutant (n=4) and control (n=4) littermates. The levels of the indicated proteins in the controls were normalized to 1.0 and the levels of the proteins in mutant samples were expressed as the relative to the normalized values. Error bars indicate s.e.m. **P<0.01 as analyzed by Student's t-test. (**b**) Quantitative analysis of the indicated uncoating related protein levels (Fig. 3d) from *kif5b* mutant (n=3) and control (n=3) littermates. The levels of the indicated proteins in the controls were normalized to 1.0 and the levels of the proteins in mutant samples were expressed as the relative to the normalized values. Error bars indicate s.e.m. **P<0.01 as analyzed by Student's t-test.

Figure S4

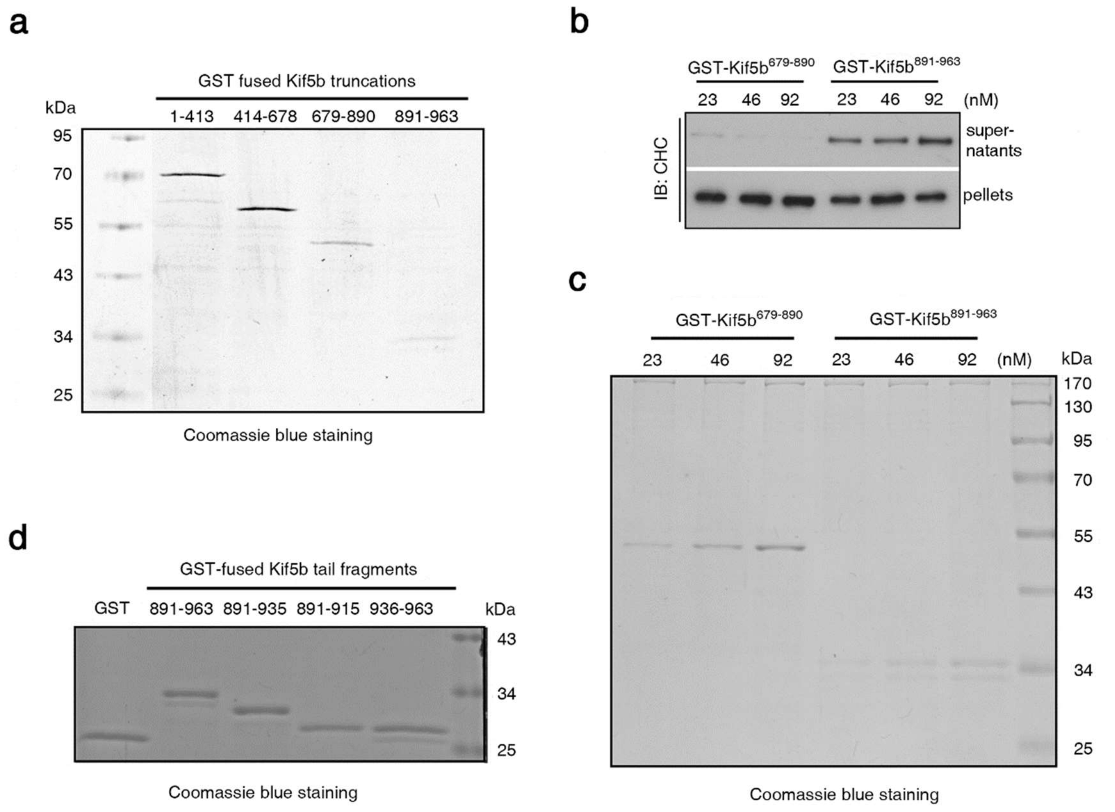

Figure S4. Kif5b tail fragment 891-963 caused a dose-dependent increase of CHC uncoating and GST-fused Kif5b fragments for CCV uncoating assay, related to Figure 3. (**a**) Coomassie blue staining of GST-fused Kif5b fragments used in the uncoating assay in Fig. 3g. (**b**) Increasing concentrations of Kif5b tail (residues 891-963) or the fragment containing the KLC-binding site (residues 670-890) were applied *in vitro* in the uncoating assay. CHC released into supernatants or remaining in the pellets was determined by Western blot analysis. (**c**) Coomassie blue staining of GST-fused Kif5b fragments used in the uncoating assay in Supplementary Fig. S4b. (**d**) Coomassie blue staining of GST-fused Kif5b fragments used in the uncoating assay in Fig. 3h.

Figure S5

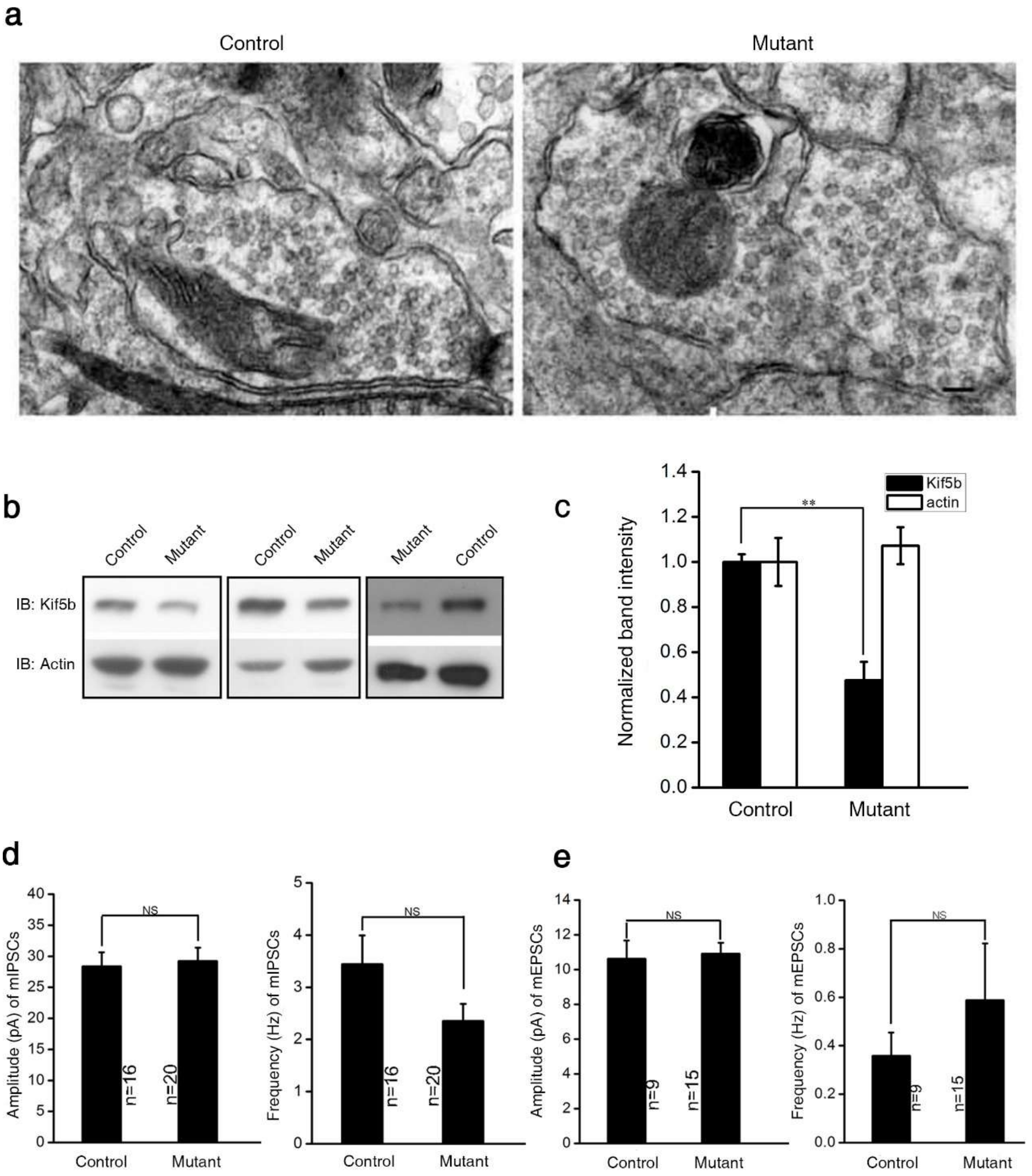

Figure S5. No alteration of synaptic transmission was caused by specific depletion of Kif5b in mouse hippocampus, related to Figure 4. (**a**) Electron microscopy examination of synapses revealed no accumulation of coated structures in *kif5b* mutant (right) compared with control (left) mouse brain samples. Scale bar=100 nm. (**b**) Representative Western blots of Kif5b in hippocampus of *kif5b* mutant mice or control littermates. Kif5b expression was remarkably down-regulated by *kif5b* knockout. (**c**) Quantitative analysis of the indicated bands in left from mutant (n=3) and control (n=3) littermates (right). The levels of Kif5b or Actin in controls were

normalized to 1.0, and the levels of the proteins in mutant samples were expressed as the relative to the normalized values. Error bars indicate s.e.m. **$P<0.01$ as analyzed by Student's t-test. (**d**) Whole-cell patch-clamp recordings of pyramidal neurons in 20-day-old mouse hippocampal sections were performed. Amplitude (left) and frequency (right) of mIPSCs from cortical sections of 20-day-old control and mutant mice. (**e**) Amplitude (left) and frequency (right) of mEPSCs from cortical sections of 20-day-old control and mutant mice.

Figure S6

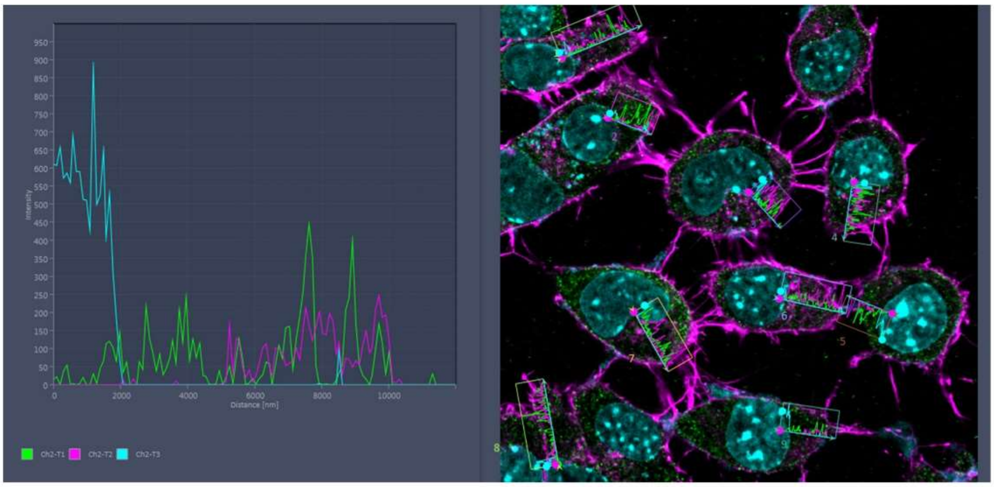

Figure S6. Representative measurements of subcellular distribution of CHC in neuro2a cells, related to Figure 3. Images were acquired by a Zeiss LSM780 confocal laser scanning microscope. The fluorescence profile was measured by the ZEN LITE (ZEISS). The subcellular distribution of CHC (Channel 1, green) was quantified by measuring the line profile of fluorescence intensity from the plasma membrane (marked by Actin staining, channel 2, Magenta) to the nucleus (marked by DAPI staining, channel 3, Cyan). One line was drawn in each cell and data were collected from 30 line profiles (30 cells) for one genotype.

Table S1. The list of proteins identified in Gel-MS/MS, related to Figure 1

Protein ID	Protein names	Gene names	Log2 LFQ intensity						-Log welch p value	MS/MS Count	Mol. weight [kDa]	Sequence coverage [%]
			IgG ctrl_1	IgG ctrl_2	IgG ctrl_3	Kif5b _1	Kif5b _2	Kif5b _3				
**Q61768**	**Kinesin-1 heavy chain**	**Kif5b**	**15.63**	**13.96**	**16.02**	**27.02**	**25.76**	**26.54**	**2.37**	**208**	**109.55**	**59.6**
Q8BGQ1	Spermatogenesis-defective protein 39 homolog	Vipas39	17.54	13.47	14.59	26.17	26.08	25.80	1.90	84	56.62	56.4
Q02819	Nucleobindin-1	Nucb1	13.29	12.96	12.80	23.99	23.80	22.99	2.99	40	53.41	41.4
Q5UE59	Kinesin light chain 1	Klc1	14.33	14.36	14.20	25.20	24.51	24.34	3.18	96	61.63	44.3
Q64464	Cytochrome P450 3A13	Cyp3a13	14.26	14.64	14.97	24.51	24.68	24.33	3.27	1	57.49	3.6
P59016	Vacuolar protein sorting-associated protein 33B	Vps33b	15.14	14.09	14.80	24.06	23.62	23.79	2.87	47	70.53	29
D3YXZ3	Kinesin light chain 2	Klc2	13.40	13.95	15.18	23.11	22.56	22.33	2.34	35	68.18	38.2
Q9DBS5	Kinesin light chain 4	Klc4	14.61	12.83	15.78	23.00	22.34	22.20	1.92	32	68.61	35.5
Q8BJA3	Homeobox-containing protein 1	Hmbox1	14.23	11.42	13.11	21.17	21.22	20.52	1.96	11	47.12	20.5
**Q68FD5**	**Clathrin heavy chain 1**	**Cltc**	**20.49**	**10.97**	**20.94**	**25.03**	**24.98**	**24.95**	**0.83**	**131**	**191.55**	**34.6**
P17426	AP-2 complex subunit alpha-1	Ap2a1	13.38	12.66	15.07	20.72	20.24	20.00	1.90	14	107.66	13.4
P84091	AP-2 complex subunit mu	Ap2m1	16.15	15.20	16.98	22.63	22.59	22.34	2.19	32	49.65	31.5
P19253	60S ribosomal protein L13a	Rpl13a;Rpl13a-ps1	14.66	14.30	14.08	20.97	20.71	20.37	2.84	5	23.46	12.8
P49117	Nuclear receptor subfamily 2 group C member 2	Nr2c2	14.59	13.68	14.48	20.71	20.44	20.12	2.54	18	65.24	16.8
Q9Z0U1	Tight junction protein ZO-2	Tjp2	16.21	13.61	14.61	20.73	20.13	20.31	1.72	16	131.28	8.6
Q61548	Clathrin coat assembly protein AP180	Snap91	13.49	13.50	14.93	19.35	19.03	19.09	2.06	7	91.85	5.3
Q6L8S8	Beta-1,4-N-acetylgalactosaminyltransferase 3	B4galnt3	13.97	12.50	15.32	19.37	18.90	18.49	1.55	2	113.51	1
P17427	AP-2 complex subunit alpha-2	Ap2a2	13.73	13.57	14.79	19.58	18.96	18.27	1.93	8	104.02	7.8
O08576	RUN domain-containing protein 3A	Rundc3a	14.48	12.87	16.84	19.73	19.55	18.95	1.24	7	50.02	7.6
P28738	Kinesin heavy chain isoform 5C	Kif5c	14.27	13.37	14.24	19.27	18.08	18.39	2.01	7	109.27	19.5
D6RIK9	Protein fantom	Rpgrip1l	13.64	13.41	14.99	19.27	17.86	18.25	1.69	1	24.00	6.6
Q9Z0E6	Interferon-induced guanylate-binding protein 2	Gbp2	14.44	14.00	13.84	17.87	20.69	17.01	1.23	1	66.74	1.9
Q9DBG3	AP-2 complex subunit beta	Ap2b1	18.87	13.53	18.35	21.05	21.07	21.54	0.89	18	104.58	11
D3YZG8	Probable bifunctional methylenetetrahydrofolate dehydrogenase/cyclohydrolase 2	Mthfd2l	14.68	13.54	13.94	18.76	18.47	17.80	1.99	1	36.44	3.3
CON__P02538			19.62	14.49	15.62	19.54	20.82	21.92	0.88	35	60.04	43.6
Q6ZQ06	Centrosomal protein of 162 kDa	Cep162	15.18	12.87	14.02	17.37	17.38	18.88	1.36	3	160.85	0.6
REV__D3Z6B1			14.86	14.59	15.30	18.28	19.19	18.28	2.00	1	47.65	0
Q3U7K7	E3 ubiquitin-protein ligase TRIM21	Trim21	14.67	12.81	14.69	18.05	17.87	17.18	1.48	6	53.33	10
P41105	60S ribosomal protein L28	Rpl28	13.11	14.62	14.41	17.08	17.86	17.73	1.66	3	15.73	16.1

Q9CZ44	NSFL1 cofactor p47	Nsfl1c	15.00	13.62	13.65	17.23	17.91	17.18	1.65	1	40.71	4.1
Q3UUI3	Acyl-coenzyme A thioesterase THEM4	Them4	14.22	13.55	15.08	17.36	17.71	17.62	1.73	2	26.03	16.1
Q8CGY8-2	UDP-N-acetylglucosamine--peptide N-acetylglucosaminyltransferase 110 kDa subunit	Ogt	14.48	13.99	15.08	17.95	17.52	17.03	1.73	5	115.73	5
Q9CQF3	Cleavage and polyadenylation specificity factor subunit 5	Nudt21	14.53	15.37	15.24	18.17	18.34	17.36	1.74	6	26.24	11.9
P27659	60S ribosomal protein L3	Rpl3	18.29	14.18	15.01	18.59	18.46	19.11	0.82	6	46.11	6.9
P01630	Ig kappa chain V-II region 7S34.1		14.24	13.93	15.46	17.48	17.47	17.25	1.58	4	12.50	21.2
P61164	Alpha-centractin	Actr1a	18.26	14.85	14.18	18.63	18.21	18.91	0.80	8	42.61	16.8
P01668	Ig kappa chain V-III region PC 7210		15.29	12.24	15.60	16.83	17.65	17.01	0.90	2	11.95	30.9
Q80YE4	Serine/threonine-protein kinase LMTK1	Aatk	14.03	12.94	13.70	16.91	16.13	15.69	1.52	2	144.61	1
Q9WV60	Glycogen synthase kinase-3 beta	Gsk3b	14.10	13.34	14.50	17.40	16.05	16.50	1.44	2	46.71	12.6
Q02257	Junction plakoglobin	Jup	14.79	13.31	14.39	17.39	16.55	16.43	1.41	10	81.80	14
Q8QZR5	Alanine aminotransferase 1	Gpt	14.72	13.64	14.58	16.65	17.12	17.06	1.71	3	55.14	4.4
Q9WUX5	Protein MRVI1	Mrvi1	14.60	12.53	15.22	18.13	15.87	15.96	0.83	1	97.43	1
Q8BFZ9	Erlin-2;Erlin-1	Erlin2;Erlin1	15.98	14.48	13.49	16.53	17.39	17.06	1.03	5	37.87	16.5
P46471	26S protease regulatory subunit 7	Psmc2	13.63	12.06	14.55	15.50	15.32	16.32	1.00	2	48.65	5.3
Q4U2R1	E3 ubiquitin-protein ligase HERC2	Herc2	13.72	14.00	15.27	16.76	17.05	15.96	1.22	2	527.45	0.8
P14131	40S ribosomal protein S16	Rps16	15.34	13.62	14.65	15.73	17.12	16.94	1.05	3	16.45	12.3
Q91XT4	Protein transport protein Sec16B	Sec16b	16.05	13.80	15.12	17.04	17.13	16.68	1.01	1	115.51	1.1
Q9EQG3	Sciellin	Scel	15.91	14.59	14.89	16.91	17.04	16.23	1.11	1	72.97	1.4
P17897	Lysozyme C-1	Lyz1	15.07	14.39	13.77	15.54	15.87	16.20	1.13	1	16.79	8.1
P62814	V-type proton ATPase subunit B, brain isoform	Atp6v1b2;Atp6v1b1	16.50	16.55	17.80	13.92	16.19	14.44	0.92	4	56.55	10.6
P80315	T-complex protein 1 subunit delta	Cct4	17.86	17.65	19.56	16.71	15.58	16.18	1.07	9	58.07	12.8
P47738	Aldehyde dehydrogenase, mitochondrial	Aldh2	16.06	16.44	16.28	14.31	13.60	14.23	1.90	2	56.54	4.8
P46660	Alpha-internexin	Ina	15.80	15.97	16.82	13.83	14.22	13.75	1.64	2	55.74	5
Q6DFY2		Opcml	16.20	16.31	16.80	13.60	14.28	14.50	1.71	2	37.16	5.9
P35278	Ras-related protein Rab-5C;Ras-related protein Rab-5A;Ras-related protein Rab-5B	Rab5c;Rab5a;Rab5b	16.81	16.23	18.31	14.97	14.33	14.89	1.18	5	23.41	22.2
P62492	Ras-related protein Rab-11A;Ras-related protein Rab-11B	Rab11a;Rab11b	15.76	15.11	17.10	14.74	12.59	12.86	0.99	2	24.39	11.1
Q3TML0	Protein disulfide-isomerase A6	Pdia6	16.18	16.06	17.72	14.69	14.76	12.20	0.96	5	48.69	13
P68368	Tubulin alpha-4A chain	Tuba4a	16.45	15.90	17.05	12.28	14.61	13.84	1.20	3	49.92	53.6
O35226	26S proteasome non-ATPase regulatory subunit 4	Psmd4	16.43	16.21	16.97	14.58	13.01	13.27	1.49	1	40.70	3.7
P18760	Cofilin-1	Cfl1	17.47	17.18	18.55	14.81	13.50	14.73	1.53	6	18.56	34.3

F7BX26	Serine/threonine-protein phosphatase; Serine/threonine-protein phosphatase 5	Ppp5c	17.44	19.52	17.30	15.08	14.20	14.42	1.35	1	54.21	2.1
P17751	Triosephosphate isomerase	Tpi1	18.64	18.47	19.64	14.37	14.52	16.91	1.25	7	32.19	23.1
P11983	T-complex protein 1 subunit alpha	Tcp1	16.96	16.33	19.41	13.49	13.95	14.29	1.20	7	60.45	17.6
Q9Z1A1		Tfg	19.95	18.22	16.85	16.43	13.83	13.72	1.00	6	43.02	21.9
Q99PT1	Rho GDP-dissociation inhibitor 1	Arhgdia	17.82	17.66	18.82	14.47	13.40	14.97	1.64	4	23.41	20.6
P97929	Breast cancer type 2 susceptibility protein homolog	Brca2	17.68	18.28	17.47	14.51	13.26	13.52	1.92	1	370.66	0.5
Q8C1B7	Septin-11	Sept11	18.21	18.36	18.41	13.80	14.16	14.60	2.47	5	49.69	10.7
P52480-2	Pyruvate kinase PKM	Pkm	18.37	17.07	21.18	14.64	14.39	14.55	1.16	9	57.98	15.1
P62259	14-3-3 protein epsilon	Ywhae	18.55	18.68	20.29	14.52	13.47	16.36	1.31	7	29.17	25.5
P10639	Thioredoxin	Txn	17.11	18.21	17.70	13.70	12.52	13.62	1.90	1	11.68	12.4
P01634	Ig kappa chain V-V region MOPC 21		17.42	18.03	18.14	14.40	12.86	13.12	1.86	2	14.90	13.2
P18525	Ig heavy chain V region 5-84;Ig heavy chain V region 914		18.87	19.52	18.31	14.26	14.68	14.18	2.15	3	12.87	23.1
O55131	Septin-7	Sept7	18.36	18.02	18.80	14.00	13.67	13.91	2.54	8	50.55	19.7
Q9R0T7		Try4;Try5	21.42	21.67	20.36	19.37	14.44	14.66	1.02	3	26.27	8.1
CON__P02768-1			18.37	18.36	18.24	12.87	13.68	12.79	2.53	2	69.37	10.3
Q61595	Kinectin	Ktn1	18.51	19.09	17.79	14.00	12.65	12.92	1.96	2	152.59	1
REV__G3UWB5			19.25	19.18	18.85	13.01	13.85	14.38	2.22	1	33.27	0
P01629	Ig kappa chain V-II region 2S1.3		19.56	19.61	19.17	13.35	14.09	14.32	2.47	1	12.22	11.6
REV__Q9Z2S7-2			21.29	21.55	20.82	13.54	13.13	14.06	2.69	1	9.04	0
